# Cerebral small vessel disease related to a heterozygous missense mutation in HTRA1: A case report

**DOI:** 10.1097/MD.0000000000044128

**Published:** 2025-08-29

**Authors:** Yuanyan Gao, Yumin Zheng, Jieren Lian, Lili Zeng

**Affiliations:** a Department of Neurology, Shanghai Civil Aviation Hospital-Ruijin Hospital Gubei Branch, Shanghai, China; b Department of Cardiology, Shanghai Civil Aviation Hospital-Ruijin Hospital Gubei Branch, Shanghai, China; c Department of Neurology and Institute of Neurology, Ruijin Hospital, Shanghai Jiao Tong University School of Medicine, Shanghai, China.

**Keywords:** CARASIL, heterozygote, HTRA1, missense mutation, small vessel disease

## Abstract

**Rationale:**

Cerebral autosomal recessive arteriopathy with subcortical infarcts and leukoencephalopathy is a rare genetic condition classified as a cerebral small vessel disease (CSVD). Traditionally, this disorder has been linked to either homozygous or compound heterozygous mutations in the high-temperature requirement A serine peptidase 1 (HTRA1) gene. Nevertheless, contemporary research has uncovered that heterozygous mutations in HTRA1 can also manifest in patients displaying patterns of autosomal dominant inheritance. In order to explore the association between the types of HTRA1 gene mutations and the genetic pattern of CSVD, in this case report, we identified a case of autosomal dominant hereditary CSVD due to a new heterozygous mutation of the HTRA1 gene in an Asian female.

**Patient concerns:**

The patient experienced a later onset of cognitive disorder and gait disturbances, and notably, there was an absence of alopecia and spondylosis, which are commonly observed extra-neurological features associated with cerebral autosomal recessive arteriopathy with subcortical infarcts and leukoencephalopathy. Neuroimaging conducted through magnetic resonance imaging revealed extensive white matter lesions and microbleeds localized within the brainstem and both cerebral hemispheres. Utilizing next-generation sequencing techniques, a novel heterozygous missense mutation in the HTRA1 gene (c.524 T>A:p.V175E) was identified.

**Diagnoses:**

The patient was diagnosed as HTRA1-related autosomal dominant CSVD.

**Interventions:**

The patient was treated with donepezil and quetiapine because of the memory impairments and visual hallucination in the early stage of the disease course. After the diagnosis of CSVD and the clinical manifestations of depressive tendencies, we treated her with Cilostazol and Sertraline additionally.

**Outcomes:**

The patient symptoms were relieved temporarily. As the disease progresses, the patient experienced 2 episodes of epilepsy and 1 cerebral infarction event.

**Lessons:**

This case suggests that individuals with the heterozygous HTRA1 mutation at V175E may also present clinical characteristics consistent with hereditary CSVD, expanding the recognized spectrum of HTRA1 mutations related to autosomal dominant small vessel disease.

## 1. Introduction

Cerebral small vessel disease (CSVD) refers to a heterogeneous group of disorders that affect the small arteries, arterioles, capillaries, and venules in the brain. Damage to these microvascular structures often leads to the onset of strokes and vascular dementia. While the majority of CSVD cases are sporadic, there are identifiable familial monogenic causes.^[1]^ These genetic forms are relatively rare, representing approximately 5% of all CSVD instances, with autosomal dominant inheritance being the most common transmission pattern.^[2]^ Among these, cerebral autosomal dominant arteriopathy with subcortical infarcts and leukoencephalopathy is recognized as the most frequent inherited variant of CSVD, primarily linked to mutations in the neurogenic locus notch homolog protein 3 gene.^[3]^ In contrast, the condition termed cerebral autosomal recessive arteriopathy with subcortical infarcts and leukoencephalopathy (CARASIL) is a recessively inherited form of CSVD, resulting from mutations in the HTRA serine peptidase 1 (HTRA1) gene. This disorder is notably characterized by a progressive decline in neurological function, early-onset alopecia, and pronounced spondylosis.^[4]^

Traditionally, CARASIL has been characterized as an autosomal recessive disorder due to homozygous mutations in HTRA1, with heterozygous mutations generally considered benign. However, recent studies indicate a potential link between heterozygous mutations in the HTRA1 gene and CSVD.^[5]^ In light of this information, we report a case of a patient exhibiting late adult-onset CSVD associated with a heterozygous missense mutation in HTRA1, who presented with dementia symptoms and a notably rapid progression of clinical manifestations.

## 2. Case report

A 60-year-old female patient of Chinese descent was referred to the neurology department at our institution due to a notable deterioration in cognitive abilities and progressive gait disturbances that had developed over the past year.

Twelve months prior to her referral, the patient began to experience challenges with maintaining balance while ambulating, alongside emotional instability and mild memory impairments, all of which occurred without significant medical intervention. In the 2 months leading up to her referral, there was a marked decline in her cognitive function. She faced difficulties in executing basic daily activities and displayed a diminished interest in activities that she previously found enjoyable. An assessment of her medical history revealed no chronic conditions such as hypertension, diabetes mellitus, or cardiovascular diseases. Importantly, she did not present with symptoms commonly associated with monogenic CSVD, such as migraines, alopecia, or lumbago. Her family medical history was largely insignificant, with all relatives reported to be in good health, with the exception of her mother, who was diagnosed with Alzheimer’s disease at the age of 85. After 2 years of post-discharge observation, the patient was found to be incapable of ambulation or performing activities of daily living independently. During the period from 2024 up to now, she had 2 epileptic seizures and got 1 stroke attack.

**Figure 1.**
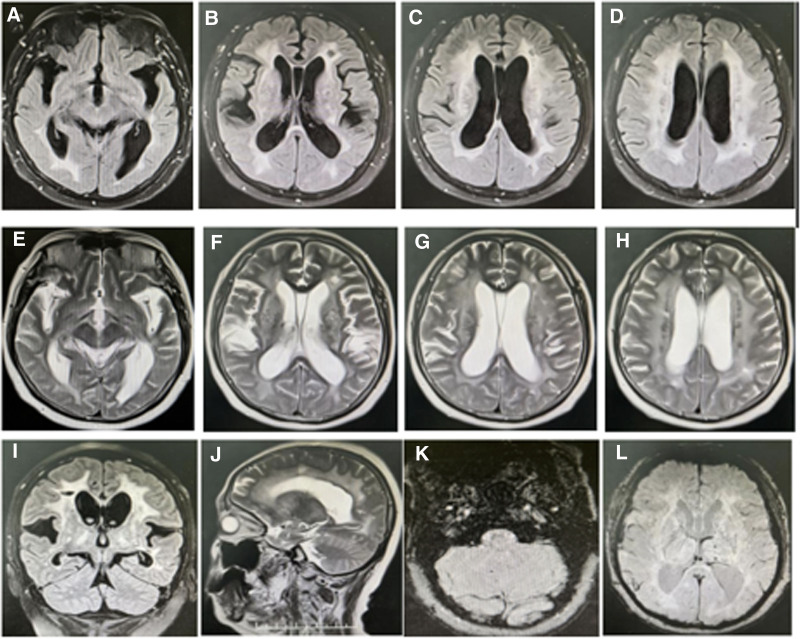
Multiple hyperintensities in the cerebral white matter extending from supra-to infratentorial regions on fluid-attenuation inversion recovery images (A–D, I) and T2-weighted (E–H, J). Susceptibility-weighted imaging reveals microbleeds in the cerebellum, subcortex, and cortex (K–L).

During a neurological evaluation, the patient exhibited a notably prolonged reaction to cognitive assessments. The Mini-Mental State Examination yielded a score of 24, whereas the Montreal Cognitive Assessment reflected a score of 21. Laboratory tests revealed significant dyslipidemia. Analyses of cerebrospinal fluid for autoimmune encephalitis, paraneoplastic neurological syndromes, and anti-glial fibrillary acidic protein antibodies returned inconclusive results. Imaging modalities, such as brain magnetic resonance imaging and susceptibility-weighted imaging (illustrated in Fig. 1A–L), identified multiple lacunar infarctions, extensive white matter lesions, and microbleeds localized within the brainstem and both hemispheres, particularly affecting the pons, thalamus, basal ganglia, as well as the periventricular and frontal-parietal-temporal-occipital regions. To enhance the diagnostic strategy, evaluations for vasculitis were performed, and magnetic resonance arterial imaging was executed, both of which yielded negative findings.

**Figure 2.**
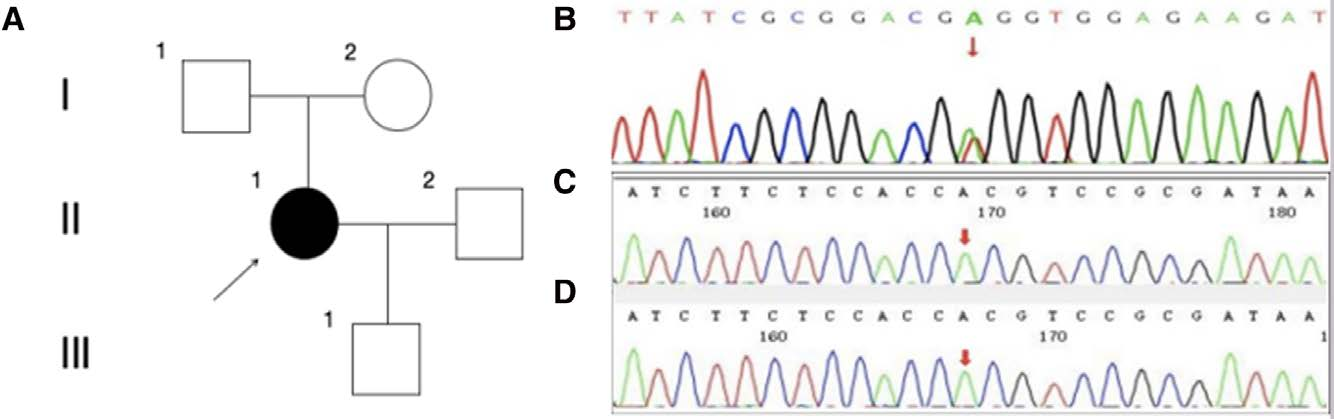
Pedigree and sequencing outcomes of the family. Family pedigree (A). Square designates male; circle indicates female; full black symbol indicates an individual affected by typical and severe clinical symptoms; empty symbol denotes a clinically healthy relative; arrow represents the proband. The heterozygous mutation of V175E in HTRA1 was identified in the patient (B). Her parents exhibited normal results (C, D). HTRA1 = high-temperature requirement A serine peptidase 1.

The family pedigree is depicted in Figure 2A. A hereditary SVD was suspected, leading to genetic testing through next-generation sequencing. This analysis uncovered a novel heterozygous missense mutation in the HTRA1 gene (NM_002775.5:exon2:c.524 T>A:p.V175E; see Fig. 2B). This specific variant was absent in reference population databases, including gnomAD. In silico evaluations utilizing 16 bioinformatics prediction tools categorized this mutation as likely detrimental to gene functionality or protein products. Notably, this mutation was not present in her parents (refer to Fig. 2C, D). This also indicates that this case of CSVD follows an autosomal dominant inheritance pattern.

## 3. Discussion

CARASIL is an autosomal recessive disorder initially documented by a Japanese researcher in 1965, later characterized by Fukutake and Hirayama in 1995. This condition is linked to homozygous mutations in the HTRA1 gene, whereas heterozygous mutations are typically considered nonpathogenic. The majority of individuals carrying heterozygous HTRA1 mutations remain asymptomatic, with only a small proportion exhibiting mild neurological symptoms. HTRA1 gene is implicated in a range of clinical manifestations. In comparison to patients with homozygous mutations, individuals exhibiting symptomatic heterozygous variations in the HTRA1 gene typically experience a delayed onset of strokes, disturbances in gait, and cognitive deterioration, while notably lacking the characteristic extracerebral symptoms that are typically associated with CARASIL.^[6]^

HTRA1 mutations can manifest in multiple forms, including heterozygous, homozygous, and compound heterozygous variants. Among the prominent phenotypes linked to mutations within the HTRA1 gene are the “classical” CARASIL syndrome, which is inherited through an autosomal recessive mechanism, and cerebral autosomal dominant arteriopathy with subcortical infarcts and leukoencephalopathy, type 2, also referred to as HTRA1-CSVD.^[7]^ The classical CARASIL syndrome is primarily caused by homozygous or compound heterozygous mutations that adhere to an autosomal recessive inheritance pattern. Conversely, HTRA1-CSVD is predominantly associated with heterozygous mutations that are transmitted in an autosomal dominant fashion.^[8]^ Both conditions are characterized by the occurrence of acute cerebral ischemic events at relatively young ages, alongside progressive dementia, gait disturbances, and mood changes. When contrasted with classical CARASIL, HTRA1-CSVD generally exhibits a milder phenotype, marked by a decreased incidence of extra-neurological symptoms such as alopecia and lumbago, as well as neurological manifestations that typically present at a later stage.^[9]^ Neuroimaging findings, which frequently include severe leukoencephalopathy, lacunar infarctions, and microbleeds, are common to both classical CARASIL and HTRA1-CSVD, however, HTRA1-CSVD typically displays less severe imaging alterations.^[10]^ In accordance with these observations, our patient did not demonstrate any signs of alopecia or lumbago but developed gait disturbances and dementia at a later age, aligning closely with the clinical characteristics of HTRA1-CSVD.

The pathophysiology underlying CARASIL is associated with homozygous or compound heterozygous mutations that lead to the functional impairment of the HTRA1 gene.^[11]^ In terms of the pathogenic mechanism related to heterozygous mutations of HTRA1, Nozaki et al^[12]^ proposed the existence of a dominant-negative effect. HTRA1 functions as a serine protease that plays a vital role in the quality control of cellular proteins. The wild-type HTRA1 protein is known to form trimers; however, the presence of mutant HTRA1 in affected heterozygotes may disrupt the activation cascade linked to HTRA1 trimer formation or impede the assembly of stable trimers. Consequently, the heterozygous variant exerts a dominant-negative effect on the proteolytic activity of wild-type HTRA1, which is essential for maintaining stable trimers.^[13]^ Additionally, the mutated HTRA1 gene results in reduced serine protease activity, leading to an inability to inhibit the transforming growth factor-beta signaling pathway. The overactivation of this pathway contributes to the accumulation of extracellular matrix, resulting in vascular intimal proliferation, disruption of the inner elastic layer, and degeneration of vascular smooth muscle, ultimately leading to ischemic and hemorrhagic events. Consequently, magnetic resonance imaging findings may reveal diffuse white matter hyperintensity, lacunar infarctions, cerebral microbleeds, and encephalatrophy.^[14]^

The HTRA1 gene, located on chromosome 10 at the locus 10q26, is responsible for encoding the high-temperature requirement serine protease. This protein comprises several distinct domains, including the insulin-like growth factor binding domain, a Kazal-like serine protease inhibitor domain, a trypsin-like serine protease domain, and a PDZ-like domain. It is noteworthy that the functioning of the HTRA1 gene is intricately linked to a variety of clinical conditions. The e protease domain can be classified into 3 distinct components: loop D (LD), loop 3 (L3), and an additional region that does not align with either LD or L3 classifications.^[15]^ Investigations have demonstrated that mutations impacting the LD or L3 domains are associated with a more pronounced adverse effect on the trimerization process in comparison to alterations occurring in non-LD or L3 regions. This ultimately leads to a decline in protease activity.^[16]^ This finding is supported by the identification of heterozygous HTRA1 mutations reported in existing literature, predominantly concentrated within the LD/L3 domain.^[10]^ In the present study, we have detected a heterozygous missense mutation, p.V175E, in the HTRA1 gene, situated near the active site of the trimer. This gene mutation was mentioned in the supplementary material of Whittaker et al,^[17]^ which has been classified as a missense variant with moderate impact, probably damaging to protein structure and deleterious to protein function. Nonetheless, it is crucial to recognize that not all patients exhibiting severe clinical manifestations carry mutations within the LD/L3 regions. The severity of the phenotypic expression is modulated not only by the specific domain of the mutation but also by vascular risk factors such as smoking and hyperlipidemia.^[10]^

In conclusion, we present a case involving a patient with HTRA1-related autosomal dominant CSVD who possesses a novel heterozygous missense mutation, c.524 T>A (p.V175E), thereby broadening the established mutation spectrum of HTRA1. Obviously, in this case, due to the limitation of the single sample size, it is impossible to represent the entire spectrum of hereditary CSVDs. Therefore, it is not possible to explore in greater depth the association between the genetic mode of CSVDs and the mutation types of the HTRA1 gene. Based on the results, we recommend that genetic testing be conducted for patients who exhibit clinical manifestations consistent with hereditary CSVD. Future studies should prioritize thorough genetic analyses and comprehensive investigations of heterozygous HTRA1 variants.

## Acknowledgments

The authors would like to acknowledge the patient and her family for their participation in the study and generously permitting the use of the data in this report. This article was edited and checked to improve to reduce the article’ s duplication rate by the helping of NewIdea generative AI.

## Author contributions

**Data curation:** Yumin Zheng.

**Methodology:** Jieren Lian.

**Supervision:** Li-li Zeng.

**Validation:** Li-li Zeng.

**Writing** – **original draft:** Yuanyan Gao.

**Writing** – **review & editing:** Yuanyan Gao.
